# Development and Application of Miniaturized Multispectral Detection System for Water Reflection Detection

**DOI:** 10.3390/s25247675

**Published:** 2025-12-18

**Authors:** Yuze Song, Yunfei Li, Chao Li, Feng Luo, Fuhong Cai

**Affiliations:** 1 National Ocean Technology Center, Tianjin 300112, China; 2 Key Laboratory of Ocean Observation Technology, Ministry of National Resources, Tianjin 300112, China; 3 School of Civil Engineering Tianjin, Tianjin University, Tianjin 300072, China; 4 College of Electronic and Information Engineering, Hebei University, Baoding 071002, China; 5 Key Laboratory of Biomedical Engineering of Hainan Province, School of Biomedical Engineering, Hainan University, Sanya 570228, China

**Keywords:** multispectral detection, water reflection detection, chlorophyll concentration calculation

## Abstract

Spectroscopic technology offers the advantage of rapid online monitoring and has attracted significant attention in molecular detection. However, the complex optical spectroscopic structure results in a relatively complex structure for spectral detection systems, limiting their widespread application. In water spectral detection, in addition to ensuring the stability of the optical system, waterproofing is also crucial. Therefore, developing miniaturized spectral detection modules in water spectral detection can improve system stability and reduce the complexity of developing and maintaining underwater hardware. This work develops a compact multispectral detection system centered on a miniature multispectral sensor. The system, controlled by a microcontroller, detects eight spectral channels within the 400–700 nm range and transmits data via the I^2^C bus. The sensitivity and stability of the detection are sufficient for water reflectance spectral detection. Based on the reflectance spectrum obtained by the above module, this work develops a regression algorithm to estimate the chlorophyll concentration in water. By comparing with standard chlorophyll concentration detection instruments, the results demonstrate the effectiveness of the proposed system in accurately estimating chlorophyll concentration.

## 1. Introduction

The global oceans cover roughly 70% of the Earth’s surface, and marine environments play a critical role in regulating climate, supporting biodiversity, and providing ecosystem services such as fisheries, carbon sequestration, and nutrient cycling. Because of this, accurate monitoring of oceanic biogeochemical variables—among which chlorophyll concentration is one of the most important—is essential for understanding ecological health, detecting pollution or harmful algal blooms (HABs), and informing policy and management. Traditional methods for monitoring chlorophyll include optical monitoring and chemical analysis [1,2,3,4]. However, these methods typically rely on off-line detection and lack the capacity for large-scale monitoring, thus limiting their use for wide-range chlorophyll surveillance. A method that can in situ monitor chlorophyll concentrations in water bodies is crucial.

Optical sensing methods have long been applied for monitoring chlorophyll in water. Instruments aboard satellites, airborne platforms, boats, or fixed stations can collect spectral reflectance or radiance information [5,6,7,8,9]. Combining imagery (spatial information) with spectroscopy (spectral information) allows for the estimation of chlorophyll concentrations. The optical sensing of chlorophyll is primarily based on two mechanisms: chlorophyll absorption and chlorophyll fluorescence. Originating from chlorophyll’s molecular structure, its absorption features can be extracted from the spectrum reflected under 400–700 nm illumination [10,11]. These absorption characteristics can then be utilized to retrieve chlorophyll concentration. The second approach relies on chlorophyll fluorescence. When excited by a light source around 400 nm, chlorophyll molecules emit fluorescence signals, whose intensity exhibits a linear relationship with concentration. Consequently, chlorophyll fluorescence also provides a direct means to estimate chlorophyll concentration [12,13,14].

Although optical sensing methods have been extensively studied in the field of chlorophyll monitoring, online chlorophyll optical detection instruments are not yet widely used, and most chlorophyll concentration measurements are still performed in laboratories. In the laboratory, both absorption spectroscopy and fluorescence spectroscopy can be derived from advanced laboratory spectrometers for precise calculation of chlorophyll concentration. However, laboratory measurements methods involve on-site sampling, sample transportation, and necessary sample preparation, which undoubtedly reduce the efficiency of chlorophyll concentration measurements. In situ chlorophyll measurement eliminates these steps, improves efficiency, and delivers more timely data on chlorophyll dynamics. This is of great significance for marine scientific research and environmental protection.

Using near-shore spectrometers for chlorophyll concentration detection is a method that is gradually being recognized as effective [15,16,17,18]. Ground-based hyperspectral imagers can be used to monitor phytoplankton blooms, cyanobacteria and water quality [19,20]. Furthermore, combining blue lasers, ground-based lidar can be used to collect the fluorescence of chlorophyll in water [21,22], thereby calculating the chlorophyll concentration at the water surface. However, due to the relatively weak fluorescence signal, these fluorescent signals can only be acquired at night when there is no sunlight or auxiliary illumination. The aforementioned ground-based spectrometers can continuously acquire chlorophyll concentration information over long periods. However, these systems are relatively complex in structure and require significant effort for daily maintenance. Designing a compact, energy-efficient, and fully functional small multispectral sensor would be far more helpful for detecting water reflectance spectra and calculating corresponding chlorophyll concentrations.

Based on the above introduction, this work employs the AS7341 multispectral sensor [23], supported by a microcontroller and PCB board, to build a stand-alone detection system capable of light source control, spectral detection, data storage, and transmission. Using this system, reflectance spectra of water with different chlorophyll concentrations were collected, and combined with regression analysis algorithms [24,25], chlorophyll inversion calculations were achieved. In this work, we focus on above-water, hand-held reflectance sensing for estimating chlorophyll, consistent with prior ground-/shore-based optical monitoring systems [26,27]. The hardware is not operated under continuous submersion. Nonetheless, the device’s miniaturized architecture reduces ingress pathways and simplifies mechanical sealing, making it a suitable basis for future splash-resistant or submersible variants.

## 2. Materials and Methods

**Figure 1:**
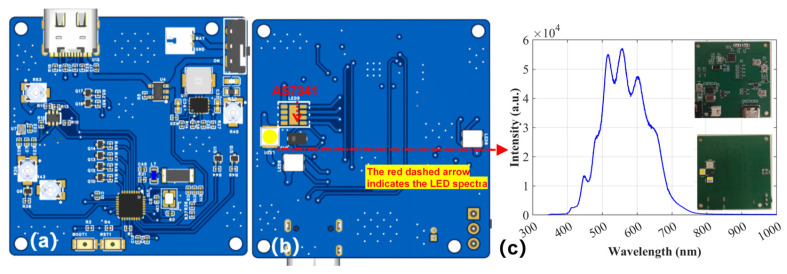
(**a**) Front and (**b**) back of PCB layout; (**c**) Spectrum of white LED; Inset: A photograph of the actual circuit board.

The hardware architecture is shown in Figure 1a,b. Its core functions are achieved through a microcontroller with the ESP32-H2FH4 microcontroller (Espressif Systems, Shanghai, China) as the main control unit, a power supply subsystem responsible for multi-voltage rail generation and battery management, a multispectral sensing subsystem for capturing accurate ambient light information, and an execution subsystem capable of independently driving various LED loads. These four subsystems communicate via an internal bus and general-purpose input/output ports, forming a complete closed-loop control platform. This allows the system to intelligently adjust the intensity of the LED light based on sensor feedback.

The ESP32-H2FH4 microcontroller, based on a low-power RISC-V architecture with integrated Wi-Fi and Bluetooth, serves as the system core controller. A 32 MHz crystal ensures stable system timing, complemented by a 32.768 kHz crystal for accurate real-time operation. The power management system supports automatic switching between USB and lithium battery inputs, providing a regulated 3.3 V main supply and a low-noise 1.8 V output for analog and multispectral sensing circuits to ensure signal stability and precision.

To achieve accurate measurement, the system integrates an AS7341-DLGM multispectral sensor (ams OSRAM, Premstätten, Austria). This sensor communicates with the main controller via an I^2^C serial bus. Given the difference between the sensor’s core operating voltage (1.8 V) and the main controller’s operating voltage (3.3 V), a pair of N-channel MOSFET are used to construct a bidirectional level shifting circuit, ensuring the correctness and reliability of cross-voltage domain communication. The system employs a custom flexible LED driving circuit, in which LEDs are controlled through N-channel MOSFETs driven by PWM signals from the main controller. This configuration enables precise digital dimming and stable illumination performance. The overall package measures 47 × 47 × 20 mm (~44 cm^3^) and is operated in air near the water surface (non-submersible); the compact form factor also facilitates future sealing and corrosion-resistant packaging. Rationale for channel selection: the eight visible bands (~415–680 nm) plus one NIR band (~910 nm) span Chl-a blue/red absorptions, the green reflectance shoulder, and red/NIR backscatter/red-edge sensitivity—capturing the main information for above-water reflectance retrieval while preserving this compact footprint and a 0.783 W power budget (Table 1). This design embodies a high degree of integration, using a single ESP32-H2 SoC (Espressif Systems, Shanghai, China) as the core to handle network connectivity, sensor data analysis, and lighting control. The drive architecture offers powerful flexibility and scalability through multiple independent MOSFETs, while the power supply design based on a buck-boost converter ensures the system’s efficiency and stability throughout its battery life. As summarized in Table 1, this footprint and the 0.783 W power draw place the instrument among the smallest and lowest-power systems, consistent with its intended above-water, hand-held use.

## 3. Results and Discussion

### 3.1. Performance Testing of the AS7341 Multispectral Sensor

**Figure 2:**
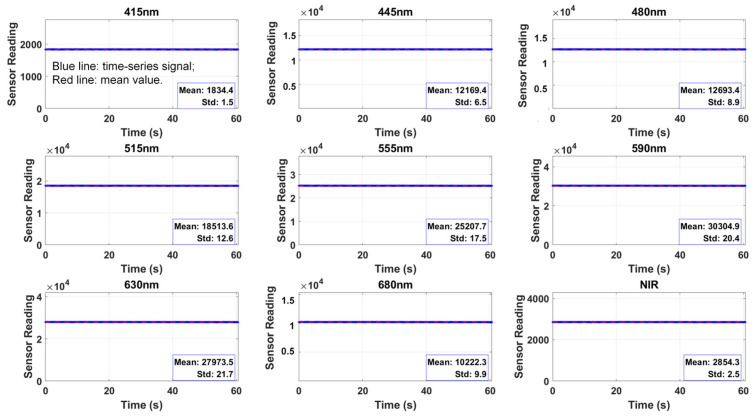
Sensing stability test of the 9 channels of AS7341 module, whose center wavelengths are 415 nm, 445 nm, 480 nm, 515 nm, 555 nm, 590 nm, 630 nm, 680 nm and 910 nm (NIR channel), respectively. The X-axis represents the time series (unit: s); the Y-axis represents the digital reading through the I^2^C port of ESP32. The statistical characteristics of the data (mean and standard deviation) are marked in the lower right corner of each subplot.

To evaluate the sensing stability of the AS7341 multi-channel detection module, continuous sampling tests were conducted on its 9 channels under constant temperature and light source conditions. Figure 2 shows the temporal stability of each channel’s signal. All channels exhibit stable outputs with minimal fluctuation and no significant drift, confirming the overall system stability. Statistical analysis showed that the stability was larger than 99%, indicating high output consistency of the AS7341 and relatively stable response characteristics among the channels. Here, the stability value greater than 99% is calculated as (mean − standard deviation)/mean, which quantifies the relative variation in the output signal. A result exceeding 99% indicates that the signal fluctuation is less than 1% of its mean, demonstrating high consistency and stability of the sensor output.

In addition to its multispectral detection capabilities, the AS7341 also features flexible adjustability. Through I^2^C commands, the device can be configured to individually set its exposure time and gain, which is crucial for spectral detection in complex lighting environments. By adjusting different exposure times and gains, it can adapt to varying lighting conditions, avoiding overexposure or weak signals. Before directly using spectra collected with varying exposure times and gain, it is essential to verify signal linearity across these parameters. Specifically, a linear response of the AS7341 readout to exposure time under constant light must be established to justify normalization by simple division and ensure data validity. This preprocessing ensures that the resulting multispectral data are directly comparable and unaffected by variations in exposure time.

**Figure 3:**
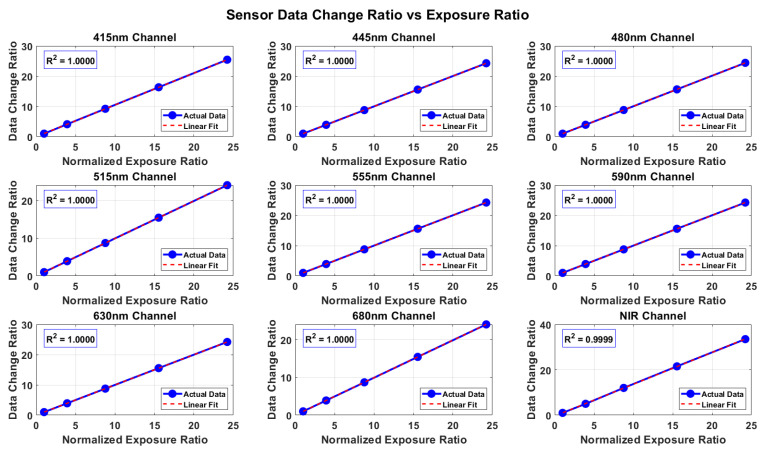
Relationship between multi-spectral signal and exposure time. A linear fit is used to display the response characteristics of each spectral channel at different exposure times.

Under constant light source intensity and temperature, the responses of the AS7341 multichannel detector under different exposure times were tested to evaluate its photoelectric conversion linearity. By gradually extending the exposure time and recording the output signal of each channel, the relationship curves between signal intensity and exposure time were obtained. The results show that the output signal of each channel exhibits a stable linear growth trend with increasing exposure time, and the overall linear fit is high (R^2^ > 0.99), as shown in Figure 3 indicating that the AS7341 maintains good linear response within the integration time adjustment range.

With uniform response across channels and linear deviations below 1%, the system maintains stable output These results demonstrate that the AS7341 detector possesses excellent exposure time linearity and time response repeatability, providing performance assurance for multi-time-series, multi-channel synchronous measurements and laying a reliable foundation for subsequent spectral calibration and dynamic optical signal measurement.

**Figure 4:**
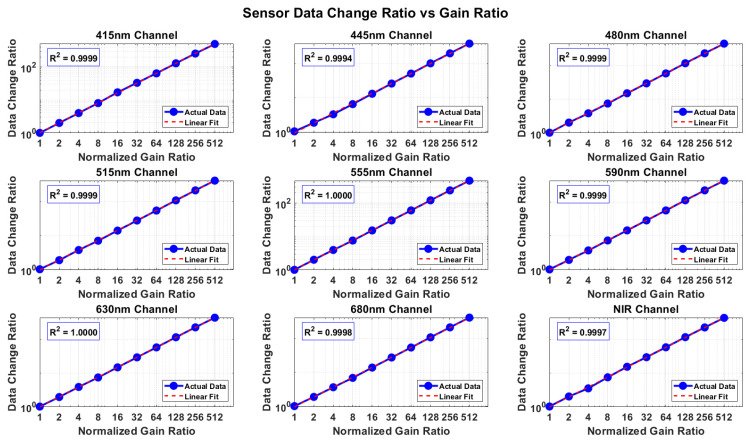
Relationship between multi-spectral signal and gain. The X-axis represents the gain factor, with gain values ranging from 1 to 512 times. The Y-axis represents the signals of each spectral channel.

Similarly, to evaluate the response characteristics of the AS7341 multi-channel detector under different gain settings, gain tests were performed on 9 spectral channels with 1×–512× gain. The results of Figure 4 shows that, except for a few channels exhibiting slight deviations, the responses of all channels demonstrate good linearity, indicating that the AS7341 module has good linear response characteristics over a wide dynamic range. Overall, the above test results verify that the sensor exhibits stable and repeatable photoelectric response characteristics in multi-channel spectral measurements, providing a reliable foundation for subsequent accurate quantitative spectral analysis.

### 3.2. Chlorophyll Concentration Calculation Based on Multispectral Reflectance Data

Water samples were collected from Dongpo Lake at Hainan University (Haidian Campus, Haikou, Hainan Province, China; coordinates: 20.06° N, 110.32° E). All samples were natural water samples and did not contain laboratory-added chlorophyll. The water samples were subjected to gradient dilution using deionized water to obtain different chlorophyll concentration levels. The chlorophyll concentration of each sample was measured using a commercial chlorophyll detector (RMD-ISY-10, Ramondo, Beijing, China) in laboratory. The measured chlorophyll concentrations for the ten gradients were approximately:5.8, 9.9, 16.2, 23.8, 33.8, 45.1, 55.0, 75.2, and 118–144 mg/L. Then, under white LED illumination, the AS7341 was used to collect the multispectral reflectance data of all water samples. All multispectral measurements were performed indoors under controlled conditions. Although only multispectral reflectance data from 9 channels can be obtained, using these multispectral reflectance data and regression algorithms [31,32,33,34], chlorophyll concentration calculation based on multispectral data can be achieved.

Specifically, to achieve accurate chlorophyll concentration retrieval under limited spectral bands, a regression framework based on the Gaussian Mixture Model–Support Vector Regression (GMM-SVR) was developed. The Gaussian Mixture Model (GMM) [35] was employed to cluster and represent the spectral samples probabilistically, thereby enhancing feature separability and capturing nonlinear spectral distributions. On this augmented feature space, a Bayesian-Optimized Support Vector Regression (BO-SVR) model was implemented to perform predictive regression. The BO-SVR algorithm integrates Bayesian optimization with traditional SVR to adaptively determine hyperparameters by constructing a Gaussian process surrogate model and iteratively maximizing the expected improvement acquisition function. Compared to conventional grid-search or manual tuning strategies, this approach efficiently explores the hyperparameter space while avoiding local minima, resulting in a more robust and generalized model performance.

**Figure 5:**
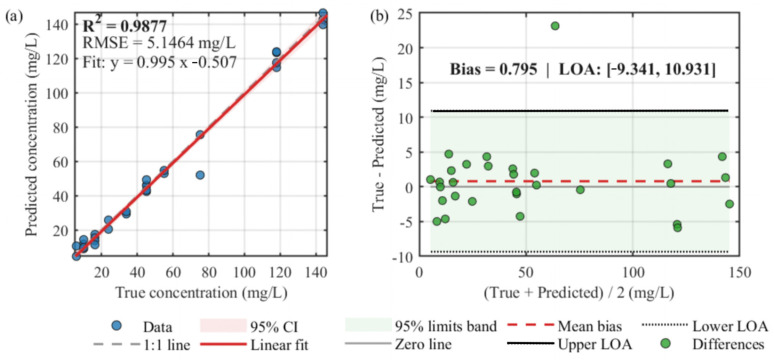
Performance evaluation of the chlorophyll concentration retrieval model based on the GMM–SVR framework optimized by Bayesian optimization (BO-SVR). (**a**) Comparison between predicted and measured chlorophyll concentrations, showing a strong linear relationship; (**b**) Bland–Altman agreement analysis between predicted and measured values.

In this work, three key hyperparameters—BoxConstraint, Epsilon, and KernelScale—were automatically optimized within a logarithmic search space. The optimal configuration obtained was BoxConstraint = 393.65, Epsilon = 0.00457, and KernelScale = 1.736, which corresponded to the best regression performance. To enhance model robustness and prevent overfitting under limited sample conditions, a 5-fold cross-validation strategy was incorporated into the regression experiments. As illustrated in Figure 5a, the predicted chlorophyll concentrations exhibited a strong linear correlation with the measured chlorophyll concentration from commercial chlorophyll concentration detector, with a determination coefficient of R^2^ = 0.9877 and a root-mean-square error (RMSE) of 5.1464 mg/L. This high degree of agreement indicates that the proposed BO-SVR model achieved reliable predictions even when trained with a limited number of spectral bands. Furthermore, the Bland–Altman consistency analysis confirmed that most prediction differences fell within the 95% limits of agreement, suggesting no significant systematic bias between predicted and observed chlorophyll concentrations. Overall, the BO-SVR-based GMM-SVR framework, combined with 5-fold cross-validation, effectively captured the nonlinear relationship between multispectral reflectance data and chlorophyll concentration, demonstrating greater model stability and generalization. The combination of probabilistic clustering and Bayesian hyperparameter optimization substantially improved regression accuracy and model generalization, demonstrating the potential of this method for high-precision chlorophyll retrieval and similar spectral inversion applications.

To rigorously evaluate the performance and highlight the distinct advantages of the proposed GMM-BO-SVR framework, a comparative analysis was conducted against several classic machine learning algorithms. These baseline models included Multiple Linear Regression (MLR) and Stepwise Linear Regression (SWLR), representing robust linear methods, as well as K-Nearest Neighbors (KNN) and a Decision Tree (DT) regressor, representing standard non-parametric and non-linear approaches.

**Figure 6:**
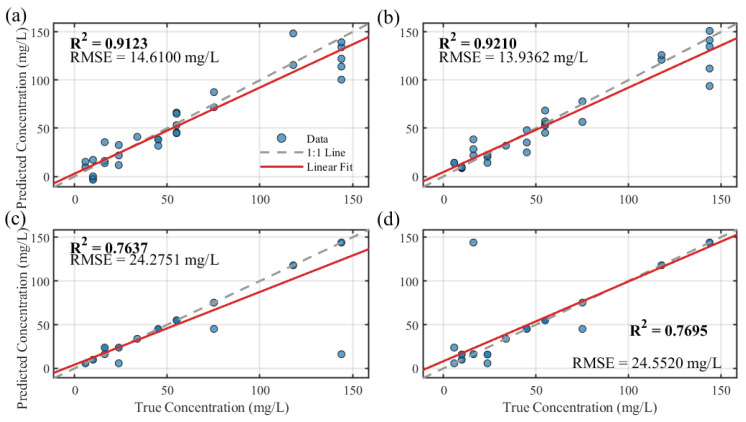
Comparative analysis of baseline regression models for chlorophyll concentration retrieval using the test set. (**a**) Multiple Linear Regression (MLR); (**b**) Stepwise Linear Regression (SWLR); (**c**) K-Nearest Neighbors (KNN, K = 5); (**d**) Decision Tree Regression (DT).

To ensure a fair and direct benchmark, all baseline models were trained and validated using the identical data partitioning (70 training samples, 30 test samples) as the primary model, utilizing the original 8-band spectral features without the GMM-based augmentation. The comparative results are illustrated in Figure 6. The MLR models exhibited moderate success; MLR achieved (R^2^ = 0.912, RMSE = 14.61 mg/L), while SWLR showed a marginal improvement (R^2^ = 0.921, RMSE = 13.94 mg/L). However, the standard non-linear models struggled to capture the complex relationships, resulting in significantly poorer fits (R^2^ = 0.764 and R^2^ = 0.770, respectively) and substantially higher errors (RMSE = 24.28 mg/L and 24.55 mg/L). The best-performing baseline model (SWLR) still produced an RMSE 2.8 times higher than that of the proposed GMM-BO-SVR framework (13.94 mg/L vs. 4.99 mg/L). This stark contrast underscores the limitations of conventional models when applied directly to the limited and complex spectral data. It simultaneously validates the efficacy of the proposed methodology, demonstrating that the combination of GMM-based probabilistic feature enhancement and Bayesian-optimized SVR is crucial for achieving high-precision chlorophyll.

### 3.3. Threshold Classification of Chlorophyll Concentration

Besides regression analysis, classifying chlorophyll concentration thresholds is beneficial for establishing early warning mechanisms in water quality monitoring. However, traditional classifiers may struggle with the non-linearity of spectral data, especially under limited sample sizes. Therefore, we propose a hybrid classification framework utilizing a multispectral sensor.

To evaluate the feasibility of classifying chlorophyll concentration from multispectral reflectance data, this work constructed and compared two machine learning models: a Support Vector Classifier with Gaussian Mixture Model (GMM) feature enhancement (GMM-SVC) and a standard Random Forest (RF). Given that the original chlorophyll concentrations were continuous values, a discretization step was first applied. We utilized the K-Means clustering algorithm (k = 5) on the one-dimensional (1D) concentration values of the entire dataset. This process partitioned the samples into five ordered, discrete classes: ‘Very Low’ (center ~13.93 mg/L), ‘Low’ (~44.63 mg/L), ‘Medium’ (~75.20 mg/L), ‘High’ (~118.00 mg/L), and ‘Very High’ (~144.00 mg/L). Subsequently, the dataset was randomly partitioned into a 70% training set (n = 70) and a 30% test set (n = 30). Given the limited sample size (N = 100) and the risk of overfitting, we employed a 5-fold cross-validation strategy within the training set to ensure model robustness.

For model development, two distinct classification pipelines were employed. The GMM-SVC approach originally included a feature enhancement step, in which a GMM was trained on the 8-band training spectra using the Bayesian Information Criterion (BIC) to automatically select the optimal number of components [31]. The computation yielded k = 1, indicating that no meaningful Gaussian subcomponents existed within the dataset. Therefore, the GMM-based probabilistic feature enhancement was not applied in this case to avoid unnecessary model complexity or potential misinterpretation. The classification was instead performed directly on the standardized 8-band spectral features using an Error-Correcting Output Codes Support Vector Classifier with a Radial Basis Function kernel.

**Figure 7:**
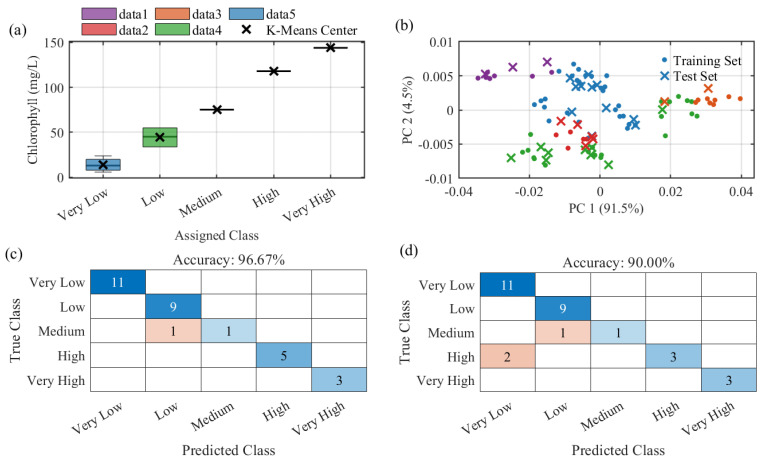
Visualization of the chlorophyll classification methodology and model performance. (**a**) Box plot illustrating the distribution of continuous concentrations within the five discrete classes defined by K-Means clustering; (**b**) Principal Component Analysis (PCA) visualization of the 8-band spectral data, colored by the five assigned classes, showing significant class overlap; (**c**) Confusion matrix for the GMM-SVC model performance on the independent test set (Accuracy = 96.67%); Color intensity indicates sample count (darker = higher); (**d**) Confusion matrix for the Random Forest (RF) model performance on the independent test set (Accuracy = 90.00%).

As a comparison, a standard RF classifier was trained on the same 8-band spectral data, configured using the ‘Bag’ method with 100 decision trees (NumLearningCycles) and a minimum leaf size of 5 (MinLeafSize) to prevent overfitting. To analyze the intrinsic data structure, a Principal Component Analysis (PCA) was first conducted on the training set [36,37] as shown in Figure 7b. The first two principal components (PC1 and PC2) explained a combined 96% of the variance (91.5% and 4.5%, respectively), yet the visualization showed severe mixing and overlap among the five concentration classes. This confirms that the relationship between chlorophyll concentration and spectral features is highly complex and non-linear, underscoring the necessity of using advanced, non-linear classification models.

The performance of both models was evaluated on the independent test set (n=30); SVC achieved 96.67% accuracy and RF 90.00% (Figure 7c,d). To compare them on the same items, we applied McNemar’s exact test (two-sided, α=0.05), which assesses paired differences in classification outcomes; the result was p=0.50.

## 4. Discussions

The multispectral detector demonstrated good stability and responsiveness under laboratory conditions; however, its applicability to complex natural waters—especially Case-2 environments (rivers, estuaries, coastal zones)—requires further investigation. Optical complexity and constituent diversity in such waters may limit the effectiveness of low-resolution multispectral sensors and associated algorithms. Although the AS7341 provides broad coverage with nine bands (400~700 nm), its spectral resolution may not fully capture subtle variations in these environments.

To address these limitations, future work will evaluate higher-resolution spectral sensors or increased channel counts to improve characterization of water-optical properties and will explore data-driven methods (e.g., CNNs, LSTMs) that can better capture nonlinear relationships as more diverse datasets become available. Beyond algorithms, the system’s miniaturized, low-power, cost-efficient design supports large-scale distributed monitoring; improving adaptability to turbidity, illumination changes, and biofouling—together with robust calibration and adaptive optical compensation—will be key for long-term deployment.

Our system is intended for shore-based, hand-held above-water reflectance measurements. (i.e., the sensor looks at the water surface under controlled illumination) rather than for continuous submersion. This approach is consistent with previous shore-based/ground-based optical monitoring methods for inland and coastal waters.

While the stability tests were conducted under idealized laboratory conditions, real-world environments may introduce additional noise, variability, and environmental factors (e.g., fluctuating temperature, lighting conditions, and turbidity) that could affect the sensor’s performance. Therefore, the results observed in the lab may not fully reflect the sensor’s behavior in more complex, natural settings.

For future field use, we plan to develop a waterproof enclosure (IP-rated per IEC 60529), apply protective coatings and corrosion-resistant materials, conduct salt-spray and biofouling tests, and validate the system in open-water environments (lakes and coastal waters) following standard procedures [38,39].

## 5. Conclusions

This study proposed and validated a compact, highly integrated multispectral detector that combines an LED driver, an AS7341 multispectral sensor, and an efficient power management module, all controlled by an ESP32 microcontroller. Despite its small form factor, the detector can flexibly acquire spectral data from nine wavelength channels (400–700 nm), with tunable exposure time and gain via the ESP32’s I^2^C interface. Laboratory experiments confirmed that the AS7341 sensor exhibits excellent stability and responsiveness under varying optical conditions.

Building upon this hardware platform, we developed a chlorophyll concentration inversion framework based on a Gaussian Mixture Model and Bayesian Optimized Support Vector Regression (GMM-BO-SVR). The GMM probabilistically clusters spectral samples to improve feature separability, while BO-SVR performs high-precision regression. Experimental results demonstrated superior prediction performance (R^2^ = 0.9877, RMSE = 5.14 mg/L) compared with traditional models such as multiple linear regression, k-nearest neighbors, and decision trees. Moreover, a GMM-enhanced Support Vector Classifier (GMM-SVC) achieved a classification accuracy of 96.67% in a five-class chlorophyll concentration task, confirming the method’s effectiveness in extracting discriminative information from multispectral reflectance data.

Overall, the integration of a low-cost multispectral sensing module with advanced probabilistic and machine learning algorithms provides a novel, scalable solution for real-time water-quality monitoring. This work lays a solid foundation for the development of intelligent, distributed sensing networks capable of bridging the gap between low-cost field detection and high-resolution optical analysis. Future studies will extend this system toward long-term field validation and multi-parameter water quality monitoring to further advance its application in environmental management and pollution control.

## Figures and Tables

**Table 1:** Comparison of compact multispectral monitoring systems.

Works	Main Controller	Dimensions (mm)	Power
Zhang et al. [28]	STM32 MCU (STMicroelectronics, Geneva, Switzerland)	>76 × 76 × 237	>5.92 W
Li et al. [29]	Embedded PC (Intel Corporation, Santa Clara, CA, USA)	300 × 160 × 160	>12 W
Nogueira et al. [30]	AS7265x (SparkFun Electronics, Niwot, CO, USA)	Sensor: 47 × 45	>1.155 W
This Work	ESP32-H2 MCU (Espressif Systems, Shanghai, China)	Sensor: 40 × 40 × 16; Overall equipment: 47 × 47 × 20	0.783 W

## Data Availability

The data presented in this study are available on request from the corresponding author.
